# Executive dysfunctions impair and levodopa improves articulatory timing in Parkinson‘s disease

**DOI:** 10.3389/fnhum.2025.1580376

**Published:** 2025-07-02

**Authors:** Elisa Herbig, Doris Mücke, Michael T. Barbe, Tabea Thies

**Affiliations:** ^1^IfL-Phonetik, Faculty of Arts and Humanities, University of Cologne, Cologne, Germany; ^2^Department of Neurology, Faculty of Medicine and University Hospital Cologne, University of Cologne, Cologne, Germany

**Keywords:** Parkinson’s disease, executive dysfunctions, speech motor control, syllable coordination, levodopa, electromagnetic articulography

## Abstract

This study investigates the effects of executive functions and levodopa on articulatory timing patterns in simple and complex syllable onsets (CV vs. CCV) in Parkinson’s disease (PD). Kinematic speech data (EMA) of 25 individuals with PD in medication-OFF as well as medication-ON condition and 25 healthy controls (HC) were recorded, and group differences were examined. Results showed preserved articulatory coordination that is skewed in time in the PD group as well as a positive effect of levodopa on these patterns. Cluster analysis revealed an age-dependent decline in executive functions across groups that correlated with the shift pattern of the second consonant in CCV sequences for the PD group. This indicates that executive dysfunctions could give rise to changes in articulatory timing patterns as the disease progresses but independently of general motor severity.

## 1 Introduction

Parkinson’s disease (PD) is the second most common neurodegenerative disorder that impacts patients’ lives on various levels. While gross motor symptoms such as bradykinesia, rigidity, resting tremor, and postural instability are most prevalent, other areas of disruption occur in the course of the disease. These, most importantly, include speech impairment in the form of hypokinetic dysarthria and non-motor symptoms such as cognitive dysfunctions (Ziegler and Vogel, 2010). PD symptoms are mainly caused by a degeneration of the cells in the substantia nigra, an integral part of the basal ganglia control circuit, that are responsible for dopamine production. The resulting dopamine deficiency affects the functionality of the basal ganglia which not only play a fundamental role in controlling motor circuits (Duffy, 2019; Ziegler and Vogel, 2010) but are also thought to be involved in cognitive processes, particularly executive functioning (e.g., Domellöf et al., 2011). However, not much is known about the relationship between speech motor control and cognitive abilities in PD so far.

Within the context of PD, speech production is particularly intriguing as it requires control over a complex set of cognitive and motor processes. Minor disruptions of individual parts or to the collaborative functionality of these processes can lead to deficits in speech output (Duffy, 2019). As a consequence of these impaired neural mechanisms, the ability to prepare and maintain speech motor programs and to switch between them is dysfunctional in individuals with PD (Spencer and Rogers, 2005) resulting in a hypo-functionality of the speech system (Thies, 2023). This manifests in some of the characteristic speech impairments in PD such as imprecise articulation in the vocal tract leading to the auditory impression of slurred speech (Ziegler and Vogel, 2010) due to reduced range, speed, and force of articulatory movements as well as rigidity of the musculature (e.g., Duffy, 2019). Accordingly, it has been found that supra-laryngeal articulation is affected in the spatio-temporal domain when compared to healthy control speakers resulting in a disruption of the inter-gestural coordination amongst others (Nip et al., 2023; Yunusova et al., 2008). However, kinematic patterns of speech production in individuals with PD still lack understanding as studies in this field are rare.

In contrast, cognitive decline as a non-motor symptom of PD is relatively well studied. It can affect memory, attention, and various levels of executive control amongst others (Aarsland et al., 2021). Executive functions are one of the most vulnerable cognitive domains in PD and deficits in this domain occur early in the course of the disease (e.g., Kalbe et al., 2016). Additionally, some researchers argue that executive functions play a crucial role in orchestrating cognitive processes and deficits in this area are therefore the underlying cause for cognitive dysfunctions in other domains (McKinlay et al., 2010). Executive functioning is commonly understood as an umbrella term that comprises various subcomponents. Among these, set-shifting has been used as an indicator of executive functioning (Sánchez-Cubillo et al., 2009) and is related to and used to measure the concept of mental or cognitive flexibility (Dajani and Uddin, 2015). In particular the impaired ability to switch between (speech) motor programs parallels findings at the cognitive level, i.e., difficulty with set-shifting or task-switching, for individuals with PD (Spencer and Rogers, 2005).

It seems evident that impairments in both motor speech production and cognitive abilities can lead to problems regarding the adaptation to communicative demands of speakers’ interactions. Deficits in this area in turn have a negative impact on the quality of life of the individuals as they consequently often perceive themselves as inadequate and tend to avoid any type of social situations amongst other things (Miller et al., 2008). However, the exact mechanisms driving these communication difficulties are still unclear, as little is known about the potential interplay between speech motor control and cognitive or linguistic dysfunctions (Smith and Caplan, 2018). A similar link has already been postulated between limb movements and cognition/executive functioning (e.g., Domellöf et al., 2011) and between acoustic parameters of speech production and task-switching skills (Thies et al., 2020).

The present study focuses on the link between cognition and motor speech functions in PD. More specifically, we investigate the interplay between cognitive executive dysfunctions and articulatory coordination in the production of syllables with low and high complexity, CV and CCV, at the kinematic level. For this purpose, we compare the timing between the tongue and lip movements during consonant and vowel production in German syllables with simple and complex onsets in individuals with PD and healthy controls (HC). The role of levodopa on these timing patterns is explored by comparing medication-OFF (med-OFF) and medication-ON (med-ON) conditions within the PD group, as it is an additional influencing factor that complicates the interplay between speech motor control and executive functioning. This is due to the variability observed when it comes to the effect of levodopa on both motor and cognitive symptoms between individuals and throughout different stages of the disease (e.g., Domellöf et al., 2011; Rohl et al., 2022). Finally, we aim to find patterns regarding the interplay between articulatory shift patterns and executive functions, incorporating additional variables such as age, motor severity, and disease duration by cluster analysis. The articulatory findings are also correlated with executive function scores, and the influence of disease duration on the results is evaluated to further define these patterns.

## 2 Materials and methods

The data analyzed in this study were collected as part of a larger research study by Thies (2023) conducted at the Department of Neurology of the University Hospital in Cologne. A subset of the data related to the production of syllables with different phonological onset parses that has not been analyzed so far is investigated here. The study was approved by the local ethics committee of the University of Cologne (18–425; date of approval was February 8, 2019). All participants gave written informed consent prior to their participation.

### 2.1 Participants

25 individuals diagnosed with idiopathic Parkinson’s disease (5 female, 20 male) aged between 40 and 77 (mean age = 60 years) and 25 age- and sex-matched healthy controls participated in the study. The individuals with PD were diagnosed between 1 to 20 years (mean = 8 years) prior to study inclusion. All participants were native speakers of German. They underwent a screening process to rule out the presence of dementia and severe depression. The Parkinson Neuropsychometric Dementia Assessment (PANDA) and the Mini Mental Status Examination (MMSE) were administered as screening tools to test for symptoms of dementia. For the PANDA, values below 14 indicate the presence of clear symptoms of dementia (Kalbe et al., 2008). For the MMSE, the cut-off was set at 19 points or below as indicating moderate to severe impairments (Folstein et al., 1975). The Beck-Depression-Inventory-II (BDI-II; Beck et al., 1996) was administered to assess depressive symptoms where scores up to 19 are interpreted as mildly depressed at most. Finally, motor functions for all participants were assessed using part III of the Unified Parkinson’s Disease Rating Scale (UPDRS; Fahn et al., 1987) where higher scores indicate more severe impairment.

To be able to investigate the influence of levodopa on syllable coordination patterns, the patients were tested by means of a levodopa challenge test (e.g., Saranza and Lang, 2021). By comparing med-OFF and med-ON conditions, this test allows for observation of the effect of a supra-maximal levodopa dosage on motor functions. Patients withheld all dopaminergic medication for at least 12 h to achieve the med-OFF state. To test the med-ON state, each patient received a predetermined oral dose of 200 mg of soluble levodopa (2 × 100/25 mg levodopa/benserazid tablets). Both motor and speech data of the individuals with PD were assessed in the OFF condition first. 30 to 40 min after levodopa intake, they were then assessed in the ON condition. The motor assessment preceded the speech recordings in each condition. Assessment by a speech therapist excluded the presence of other speech and language problems such as aphasia, apraxia of speech, or developmental disorders. Dysarthria severity was rated as mild to moderate for all patients.

### 2.2 Neuropsychological assessment

All participants underwent a neuropsychological assessment of executive functions. Patients were only tested in med-ON condition to evade effects of motor deficits on task performance that are usually present during med-OFF condition. The *Trail Making Test* (TMT; Reitan, 1992) is not only one of the most widely used but also one of the most reliable neuropsychological assessments to measure executive functions (Sánchez-Cubillo et al., 2009; Strauss et al., 2006). It is said to capture various cognitive abilities such as processing speed, mental or cognitive flexibility (including set-shifting), visual-motor skills, or attention (Bowie and Harvey, 2006; Strauss et al., 2006). The TMT consists of two parts (A and B). In part A, the participants are asked to connect a sequence of consecutive numbers from 1 to 25. In part B, the participants are asked to connect a sequence of numbers and letters, alternating between the two (i.e., 1-A-2-B etc.). The time needed to complete each part individually is measured in seconds whereby errors made are corrected by the administrator right away without stopping the timer. The time needed to correct an error is thereby added to the total score. If a participant cannot complete part A, a maximum score of 180 s is given. If they fail to complete part B, a maximum score of 300 s is given (Strauss et al., 2006).

The literature is not entirely unanimous about the different measures and corresponding cognitive abilities derived from the TMT. However, the following correspondences have reached some acclaim and will serve as a basis for the analysis of the data presented here. TMT[A] serves as an indicator for processing speed (visual search and motor speed), while performance on TMT[B] mainly allows for drawing conclusions on mental flexibility. Apart from these pure scores of the two parts of the TMT, difference (TMT[B-A]) and ratio (TMT[B/A]) scores can provide more nuanced information on cognitive abilities. Both measures are an attempt to control for influences of motor control and other non-set-shifting elements such as visual search or working memory, which seem to play into both parts of the TMT to a certain extent, thereby emphasizing set-shifting abilities and consequently executive function (e.g., Muir et al., 2015; Sánchez-Cubillo et al., 2009). All four measures ([A], [B], [B-A], [B/A]) will be calculated to compare the cognitive abilities of individuals with PD and HC in the present data. The focus will be on the scores involving TMT[B] in order to be able to draw inferences on executive functioning in individuals with PD.

### 2.3 Speech recording and speech material

Acoustic and kinematic speech data were recorded using a 3-dimensional electromagnetic articulograph (EMA, AG 501, Carstens Medizinelektronik). For the acoustic recordings, a condenser microphone headset (AKG C 544 L, 44.1 kHz/16 bit) was used to keep the mouth-to-microphone distance of 7 cm constant. To track the articulatory movements, EMA sensors were placed on the lower lip for labial consonants (/p/), the tongue tip for alveolar consonants (/l/), and on the tongue body for dorsal vowels (/i/). They were attached to the articulators by using tissue adhesive. The tongue tip sensor was placed approximately 1 cm and the tongue body sensor approximately 4 cm from the actual tip of the tongue to ensure minimal obstruction during articulation. Two additional sensors behind the ears functioned as reference points for later correction of head movements. The raw data were converted into positional data first and then head-corrected and rotated into a head-based coordinate system using standardized bite plane measures and the respective software package provided by Carstens.

The speech material consisted of words with simple and complex onsets with initial syllables of the target words following either a simple CV (either C_1_V /pina/ or C_2_V /lina/) or complex CCV (C_1_C_2_V /plina/) structure. The stimuli were embedded in the carrier phrase “Er hat wieder [TARGET] gesagt” (“He said [TARGET] again”) (cf. methods in Hermes et al., 2019). The participants had to produce each stimulus twice in a randomized order, alternating with other cluster combinations that are not analyzed in this paper. Consequently, the following amount of target word productions were supposed to be recorded: 25 healthy controls x 3 target words x 2 repetitions = 150, 25 individuals with PD x 2 conditions (med-OFF & med-ON) x 3 target words x 2 repetitions = 300. Participants were asked to repeat the phrase if accidental speech errors, such as mispronunciations, disfluencies, or reading errors, occurred. In case participants repeated items, all intelligible repetitions were included in the analysis. This sometimes led to more than the anticipated six data points per speaker. Target words that were not clearly identifiable due to unclear pronunciation or a slip of the tongue were excluded from the analysis. Single renditions where odd data trajectories were noticeable, probably due to issues with a sensor, were also excluded. The data of four HC speakers had to be entirely excluded from the analysis due to issues with the sensors during recording that led to inaccurate data trajectories or due to incorrect articulation of the target words. In the end, 131 items for the HC speakers and 293 items for the individuals with PD (143 for med-OFF, 150 for med-ON) were included in the analysis.

### 2.4 Speech data annotation and measurements

Speech data were processed in the EMU-webAPP of the EMU-SDMS environment (Winkelmann et al., 2017). The first step was the acoustic annotation of the target words and the relevant segments in the respective first stressed syllables (C_1_ /p/ and/or C_2_ /l/, and V /i/) according to the speech waveform and the wide-band spectrogram. The stop segments, /p/, were annotated from the beginning of the oral closure to the end of the consonantal segment including burst and/or aspiration phase, i.e., from the beginning of the energy gap to the end of the aperiodic noise in the acoustic signal. Segmental landmarks for the lateral, /l/, were labeled at the abrupt change in the amplitude of the second formant as a phonetic characteristic of the lateral airflow. Lastly, segmental boundaries for /i/ were identified with respect to the periodic formant structure of the second formant.

**Figure 1:**
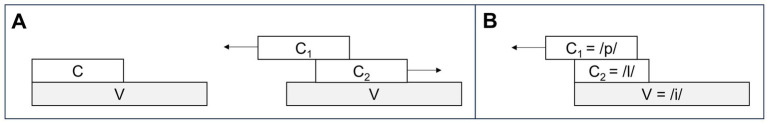
**(A)** Articulatory timing patterns of CV syllables (left), C_1_C_2_V syllables exhibiting C-center effect (right), and **(B)** the asymmetrical pattern for /pl/ in German.

Kinematic measurements were conducted within the framework of Articulatory Phonology (AP) - a dynamical approach to phonology integrating planning and performance of a language into one system. Within AP, speech can be decomposed into articulatory gestures that overlap in time, such as a lip closure during a bilabial stop (/p/) or a tongue tip closure during an alveolar lateral (/l/). On the level of prosodic units, syllable structure is assumed to emerge from stable timing relations between consonants and vowels (Iskarous and Pouplier, 2022; Yunusova et al., 2008). The timing patterns are attributed to a theory of lexically specified coupled oscillators that trigger and constrain gestural initiation in simple and branching syllable onsets. In order to uncover the phonological orchestration of syllable structure, kinematic measurements can be applied to articulatory trajectories. Traditionally, the kinematic measurements are focused on timing relations between target-to-target achievements of consonant and vowel productions (i.e., maximum achievements of linguistic constrictions in the vertical dimension) that show a higher stability than highly context-dependent onset-to-onset measures (e.g., Browman and Goldstein, 2000; Hermes et al., 2017; Hermes et al., 2019; Iskarous and Pouplier, 2022; Kühnert et al., 2006). For CV and CCV syllables, the following overlap pattern are assumed as being part of the speaker’s grammar: In syllables with a simple onset (CV), the consonantal and vocalic movements are supposed to be initiated at the same time, but due to its gestural specification, the vocalic movement is parametrized as being slower and longer than the consonant. This leads to the impression of a CV sequence on the acoustic surface (Figure 1A, left). In syllables with a complex onset (C_1_C_2_V), these timing patterns are phonologically reorganized to show C-center coordination. In the C-center coordination, C_2_ is expected to shift toward V and C_1_ shifts away from V (Mücke, 2018; Figure 1A, right). This shift pattern leads to the compression of segments on the acoustic surface. However, it has been shown for West Germanic and Romance languages that coarticulatory synergies systematically affect the symmetry of the timing pattern in /pl/−onsets (Brunner et al., 2014; Pouplier, 2012). Since the jaw is already high in /p/, the movement of the tongue tip to reach its target for /l/ can be expected to be shorter. This is described as a biomechanical shortening effect that blocks the rightward shift of C_2_. It is reflected by an asymmetrical shift pattern in the kinematic dimension of syllable production in German /pl/−onsets (Mücke et al., 2020; Figure 1B). In addition, biomechanical shortening would also lead to lower peak velocities during the production of C_2_ /l/ in complex syllables compared to respective peak velocities in simple syllables (see Mücke et al., 2020 for younger and older speakers of German).

**Figure 2:**
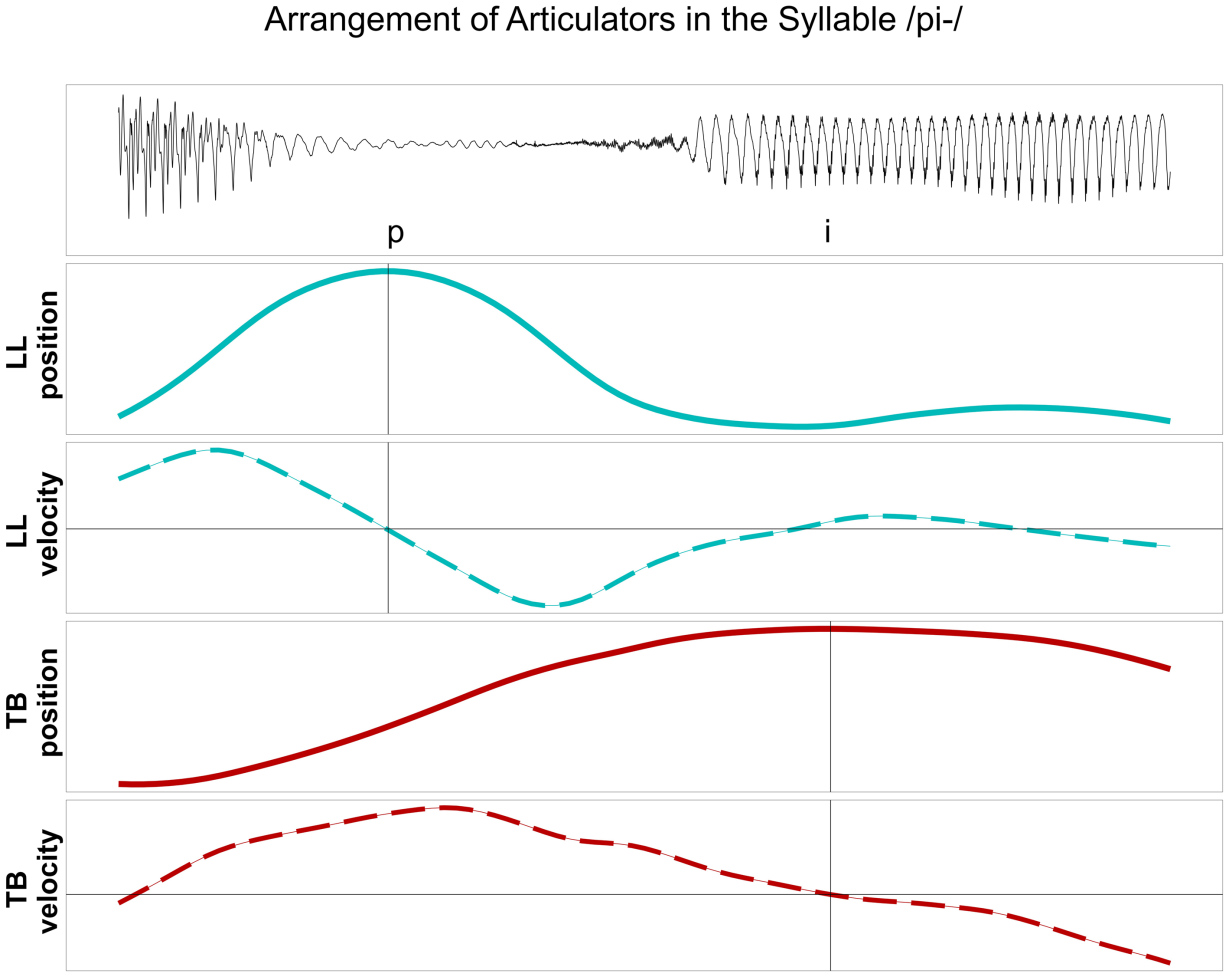
Lower lip (LL) and tongue body (TB) movements, their vertical position and respective velocity, during the production of /pi/ produced by a healthy control speaker. Target position of the articulator is aligned to zero-crossing in the velocity trace.

**Figure 3:**
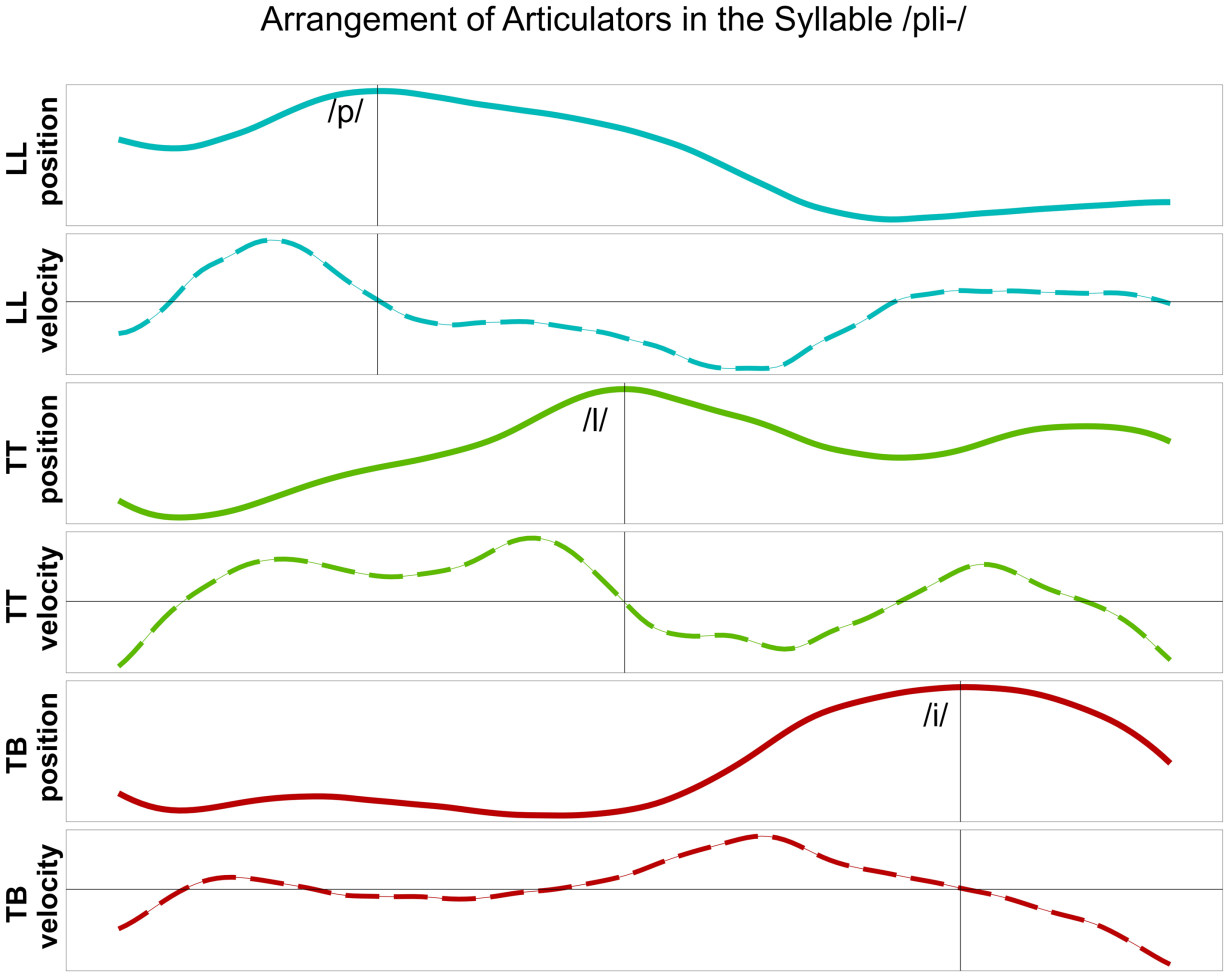
Lower lip (LL), tongue tip (TT) and tongue body (TB) movements, their vertical position and respective velocity, during the production of /pli/ produced by a healthy control speaker. Target position of the articulator is aligned to zero-crossing in the velocity trace.

In line with previous studies (Hermes et al., 2017; Hermes et al., 2019; Kühnert et al., 2006), the analysis at the kinematic level is focused on the timing patterns between overlapping articulatory movements measured in the vertical dimension (constriction degree), i.e., the maximum raising and lowering of the articulator, reflecting the articulatory goal. The C-center measure involves a purely temporal measure of stability that is based on temporal lags between the consonantal and vocalic targets (Iskarous and Pouplier, 2022). Kinematic targets do not necessarily correspond to segmental boundaries, i.e., gestural orchestration patterns cannot be captured by segmental boundaries in the acoustic signal. In line with the relevant literature, a kinematic target landmark was defined for each articulatory movement by means of the zero-crossing in the respective velocity trace: the maximum target of the movement, i.e., the point of maximum constriction. Figure 2 displays an example from a healthy control speaker for vertical movements and respective velocity of the lower lip (LL) and the tongue body (TB) in the target syllable /pi/. Figure 3 illustrates an example of a healthy control speaker for vertical movements and respective velocity of the LL, the tongue tip (TT), and the TB in the target syllable /pli/. High values in the positional curves indicate a raising of the articulator, while low values indicate a lowering. Zero crossings in the velocity trace mark the point in time when the maximum target in the positional curve is achieved. The latencies between the different landmarks inform the degree of overlap between them: From simple CV syllables, with either C_1_ or C_2_, to complex C_1_C_2_V syllables, the latency between C_1_ and V is expected to increase (overlap decreases as C_1_ shifts to the left) while the latency between C_2_ and V is expected to stay roughly the same (overlap does not change significantly as C_2_ does not shift to the right for /pl/ in German) (Mücke, 2018; Pouplier, 2012).

The following parameters were extracted and calculated by means of the “emuR” (Jochim et al., 2024) package in the software R (version 4.3.3; R Core Team, 2024). At the acoustic level, segment durations for C_1_, C_2_, V, and the entire CV and CCV syllables were calculated. At the articulatory level, latencies between the consonants and the vocalic target were extracted for both syllable types. For simple CV syllables: (1) latency between the target of the prevocalic consonant (C_1_ for /pina/, C_2_ for /lina/) and the vowel (V) were extracted. For complex CCV syllables: (2) latency between the target of the leftmost consonant (C_1_) and the vowel (V), (3) latency between the target of the rightmost consonant (C_2_) and the vowel (V) were extracted. Consonantal shifts were calculated by comparing the latencies in C_1_V/C_2_V and C_1_C_2_V syllables: The (per speaker) mean values of (2) or (3) were subtracted from the respective mean values of (1). Further, we determined the value of the highest peak velocity of the TT movement for the production of the alveolar lateral /l/ in CV and CCV syllables (Figure 3).

### 2.5 Statistical analysis

The data were analyzed using the statistical computing software R (version 4.3.3; R Core Team, 2024). To test differences in acoustic segment durations, articulatory timing patterns, and peak velocities between syllable structures (CV vs. CCV) and between groups/conditions (HC vs. PD med-OFF, HC vs. PD med-ON, PD med-OFF vs. PD med-ON), linear mixed effect models were conducted by using the lme4 package (Bates et al., 2015). Syllable structure and group/medication condition were set as predictor variables and and by-speaker random intercepts were included. Additionally, when testing differences in vowel and syllable durations, random intercepts for the critical word, as both /pina/ and /lina/ correspond to simple syllable structure, were included. The predictor effect was validated by comparing a full model (with both predictors) to a reduced model (without the critical predictor) via ANOVA. If it was found significant at *p* < 0.05, pairwise post-hoc analyses were conducted by using the emmeans package with the Tukey method (Lenth, 2024). We did not model interaction effects between the predictor variables as they are categorical in nature (syllable structure: either CV or CCV; group/medication condition: either HC, PD med-ON, or PD med-OFF) and because the same effects of syllable structure are expected for the groups/conditions under investigation.

A linear regression model was fitted to investigate group differences regarding the executive function scores with group (HC or PD med-ON) as predictor variable. The predictor effect was validated via ANOVA, comparing a model with the critical predictor “group” to a model without the predictor variable. If it was found significant at *p* < 0.05, pairwise post-hoc analyses were conducted by using the emmeans package with the Tukey method (Lenth, 2024).

We performed an exploratory cluster analysis to understand our data outcome and to organize it into groups based on a set of variables of interest and similarities in their behavior. Parameters that were considered for the cluster analysis are: C_2_ shift measures, TMT scores, speakers’ age, BDI, PANDA, and MMST for both groups as well as UPDRS and disease duration for the PD group. First, continuous variables were standardized before a cluster classification was created using the k-means clustering method. This was carried out separately within the PD and HC groups as well as across the two groups. The number of clusters was determined by visual inspection using the factoextra package with either the wss or silhouette method (Kassambara and Mundt, 2020). To interpret and characterize the clusters, we wanted to understand which variables differ across them. Thus, linear regression models were fitted to investigate cluster differences regarding the parameters listed above with cluster as a predictor variable. Again, the predictor effect was validated via ANOVA, and if it was found significant at p < 0.05, pairwise post-hoc analyses were conducted by using the emmeans package with the Tukey method (Lenth, 2024).

Additional correlation analyses were carried out for the PD group: the difference in C_2_ shift measures between CV and CCV syllables was correlated with the different TMT scores, UPDRS, and disease duration. The Shapiro–Wilk test was applied to assess whether the data in question is normally distributed (*p* > 0.05) or not (*p* < 0.05). If it turned out to be normally distributed, the Pearson method was used to examine whether articulatory timing patterns are associated with any of the tested variables. If it was found not to be normally distributed, the Spearman method was used.

## 3 Results

### 3.1 Syllable coordination: acoustic and kinematic data

The mean acoustic segment durations in milliseconds (ms) as well as sd values for C_1_ /p/, C_2_ /l/, V /i/, and for the entire syllable are reported in Table 1. Results of the statistical analysis show that durations of C_1_ /p/ differ neither between syllable structures nor between groups/conditions (*p* > 0.05 across all comparisons). C_2_ durations are shorter in complex compared to simple syllables due to coarticulatory compression (HC vs. PD med-ON: *p* < 0.001, mean difference = 52.9 ms | HC vs. PD med-OFF: *p* < 0.001, mean difference = 51.1 ms | PD med-OFF vs. PD med-ON: *p* < 0.001, mean difference = 64.5 ms). The comparison between groups/conditions shows longer C_2_ durations for the PD med-OFF group compared to the HC group (*p* = 0.012, mean difference = 21.3 ms). The durations decrease from PD med-OFF to PD med-ON, eliminating group differences between PD med-ON and HC (p > 0.05). V durations do not differ between syllable structures across all groups/conditions (p > 0.05). However, PD med-OFF presents with longer V durations both compared to HC (*p* = 0.010, mean difference = 28.5 ms) and to PD med-ON (*p* < 0.001, mean difference = 16.1 ms). The durations decrease from PD med-OFF to PD med-ON, eliminating group differences between PD med-ON and HC (*p* > 0.05). The addition of a second consonant in complex CCV onsets leads to generally longer syllable durations. When comparing groups/conditions, it can be deduced that longer durations of C_2_ and V in PD med-OFF lead to longer syllable durations in this condition.

**Table 1:** Mean acoustic segment durations in milliseconds specified by group/condition and by syllable structure.

Group/condition	Syllable structure	/p/	/l/	/i/	syllable
HC	C_1_V	197 (64)	—	121 (37)	318 (90)
	C_2_V	—	96 (34)	133 (37)	230 (64)
	C_1_C_2_V	205 (78)	57 (24)	117 (36)	379 (110)
med-ON	C_1_V	188 (45)	—	136 (40)	324 (67)
	C_2_V	—	121 (68)	144 (43)	265 (94)
	C_1_C_2_V	186 (62)	55 (20)	123 (31)	364 (86)
med-OFF	C_1_V	200 (44)	—	150 (47)	350 (77)
	C_2_V	—	130 (57)	166 (48)	296 (89)
	C_1_C_2_V	197 (68)	67 (23)	142 (54)	406 (106)

Sd values in brackets.

**Figure 4:**
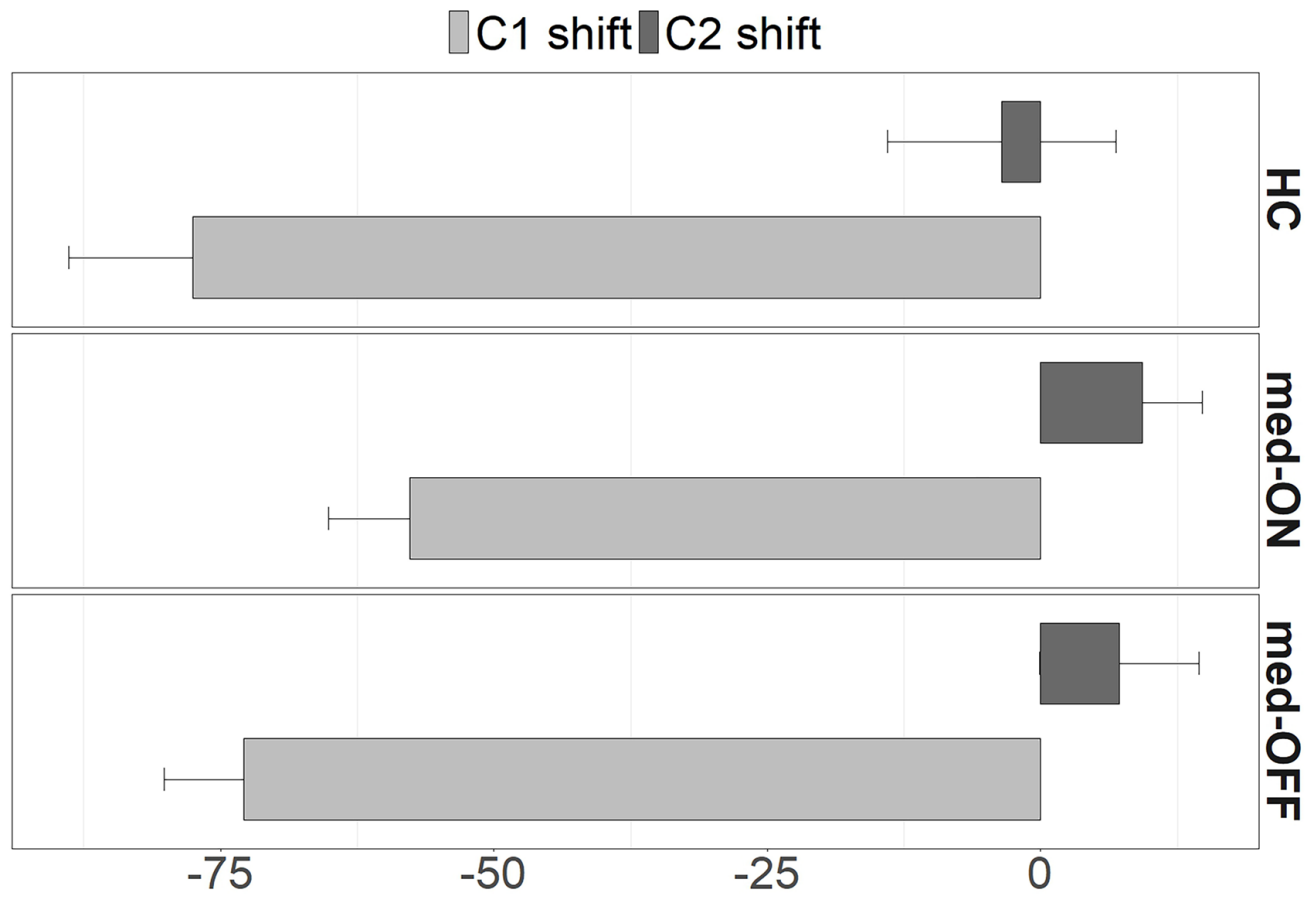
Shifts of C_1_ (light grey) and C_2_ (dark grey) from CV to CCV syllables in milliseconds for HC, PD med-ON, and PD med-OFF. Shift direction: < 0 to the left, > 0 to the right. SE represented by whiskers.

Moving on to the kinematic level, the mean articulatory latencies in milliseconds (ms), and corresponding sd values, between C_1_ /p/ and the vocalic target as well as between C_2_ /l/ and the vocalic target are reported in Table 2. Latencies from C_1_ to V increase from simple to complex syllables (HC vs. PD med-ON: *p* < 0.001, mean difference = 66.7 ms | HC vs. PD med-OFF: *p* < 0.001, mean difference = 74.9 ms | PD med-OFF vs. PD med-ON: *p* < 0.001, mean difference = 64.3 ms), reflecting a leftward shift of C_1_ away from the following vowel to make room for the added C_2_ in CCV. When comparing groups/conditions, latencies of C_1_ to V are longer in PD med-OFF compared to PD med-ON (*p* < 0.001), mean difference = 20 ms). When looking at the mean values, latencies tend to be 19.6 ms longer on average for PD med-OFF compared to HC. Latencies from C_2_ to V differ between CV and CCV syllables in the two PD conditions only, i.e., they are longer in C_2_V compared to C_1_C_2_V (*p* = 0.026, mean difference = 9.73 ms). When comparing groups/conditions, the C_2_ latencies are longer in PD med-OFF both compared to HC (*p* = 0.016, mean difference = 30.8 ms) and compared to PD med-ON (*p* < 0.001, mean difference = 15.8). Yet again, the latencies decrease from PD med-OFF to PD med-ON, eliminating group differences between PD med-ON and HC (*p* > 0.05). The resulting shift pattern for the complex onset /pl/ for the different groups/conditions is visualized in Figure 4. It shows the leftward (negative values) and rightward (positive values) shifts of C_1_ and C_2_, respectively. The peak velocities for the movement producing the alveolar lateral /l/ are reported in Table 3. Peak velocities do not differ between the groups nor between conditions but in general between CV and CCV syllables. Slower peak velocities are found in CCV syllables (HC vs. PD med-ON: *p* < 0.001, mean difference = 21.4 mm/s | HC vs. PD med-OFF: *p* < 0.001, mean difference = 23.6 mm/s | PD med-OFF vs. PD med-ON: *p* = 0.010, mean difference = 11.4 mm/s).

**Table 2:** Mean articulatory latencies in milliseconds specified by group/condition and by syllable structure.

Group/condition	Syllable structure	C_1_ to V /p/ → /i/	C_2_ to V /l/ → /i/
HC	C_1_V	177 (63)	—
	C_2_V	—	119 (51)
	C_1_C_2_V	254 (75)	119 (47)
med-ON	C_1_V	188 (52)	—
	C_2_V	—	141 (42)
	C_1_C_2_V	243 (53)	130 (38)
med-OFF	C_1_V	201 (50)	—
	C_2_V	—	158 (55)
	C_1_C_2_V	274 (68)	147 (52)

Sd values in brackets.

**Table 3:** Mean peak velocities of /l/ in mm/s specified by group/condition and by syllable structure.

Group/condition	Syllable structure	Mean peak velocity /l/
HC	C_2_V	120 (76)
	C_1_C_2_V	84 (69)
med-ON	C_2_V	112 (44)
	C_1_C_2_V	101 (40)
med-OFF	C_2_V	105 (53)
	C_1_C_2_V	97 (47)

Sd values in brackets.

### 3.2 Executive functions

Mean TMT performance scores in seconds (s) and values for the two derived scores are shown in Table 4. TMT[A] differs between the two groups, with the PD med-ON group taking longer to complete the test compared to the HC (*p* = 0.042, mean difference = 8.28 s). When looking at the mean values of TMT[B] and the derived scores, the same tendency is observable with the PD med-ON group presenting with slightly longer performance time values.

**Table 4:** Mean TMT performance scores in seconds and derived scores specified by group.

Group/condition	TMT[A]	TMT[B]	TMT[B-A]	TMT[B/A]
HC	30.3 (10.2)	78.1 (31.3)	47.8 (28.4)	2.67 (0.9)
med-ON	38.8 (14.9)	94.1 (61.6)	55.3 (54.4)	2.42 (1.03)

Sd values in brackets.

### 3.3 Cluster analysis

One speaker in the HC group was excluded from the cluster analysis, as this person did not complete the TMT. Additionally, one speaker in the PD group was excluded, as they had obtained a maximum score on the TMT[B] and appeared as an outlier in the cluster analysis, i.e., the person was assigned into a separate cluster made up of only this one data point. Besides C_2_ shift, age, TMT[A], TMT[B], TMT[B-A], and TMT[B/A], scores for BDI, PANDA, and MMST were also included in the cluster analysis. However, the latter three are not reported, as they were not significantly different between clusters for all three performed sub-analyses (across groups, HC only, PD med-ON only). Disease duration and UPDRS scores were included in the analysis for better characterization of the PD group in relation to cluster classification and classifying parameters.

The characteristics of articulatory C_2_ shift, age, executive function scores, and, if relevant, disease duration and UPDRS for all clusters and sub-analyses are shown in Table 5. Across both groups, the cluster analysis revealed two clusters. Cluster 1 comprises 34% of all participants and is characterized by older age and higher scores on both parts of the cognitive TMT test as well as higher derived scores. The C_2_ shift does not differ between the two clusters. For the HC group, the analysis revealed two clusters. Paralleling what is found across groups, cluster 1, which comprises 45% of the HC, is characterized by older age and higher scores on TMT[A], [B], and [B-A]. Again, the C_2_ shift does not differ between the two clusters. For the PD med-ON group, the analysis revealed two clusters. Cluster 1 comprises 46% of the individuals with PD and is characterized by more extreme rightward shifts of C_2_, older age, higher TMT[A], [B], and [B-A] scores as well as longer disease duration.

**Table 5:** Cluster characteristics for C_2_ shift (in milliseconds), age (in years), TMT[A], TMT[B] (in seconds), TMT derived scores specified for all three sub-analyses + disease duration (in years) and UPDRS for PD group only.

Group/condition	Variable	Cluster 1	Cluster 2	*p*-values
Across groups	C_2_ shift	17.3 (23.5)	−3.2 (42.9)	*p* > 0.05
	age	66.9 (4.9)	56.8 (6.6)	*p* < 0.001 *
	TMT[A]	44.5 (15.0)	29.5 (9.2)	*p* < 0.001 *
	TMT[B]	117.2 (29.1)	61.8 (14.4)	*p* < 0.001 *
	TMT[B-A]	72.7 (27.9)	32.3 (14.1)	*p* < 0.001 *
	TMT[B/A]	2.8 (1.0)	2.2 (0.7)	*p* = 0.02 *
HC	C2 shift	−8.6 (31.6)	3.3 (60.0)	*p* > 0.05
	age	66.1 (5.3)	54.4 (5.4)	*p* < 0.001 *
	TMT[A]	36.1 (9.4)	25.9 (9.4)	*p* = 0.03 *
	TMT[B]	100.7 (31.8)	58.3 (11.9)	*p* < 0.001 *
	TMT[B-A]	64.6 (32.5)	32.4 (11.9)	*p* = 0.01 *
	TMT[B/A]	2.9 (1.1)	2.4 (0.6)	*p* > 0.05
PD med-ON	C_2_ shift	25 (22.8)	−5.2 (23.7)	*p* = 0.005 *
	age	66.7 (5.1)	55.6 (5.5)	*p* < 0.001 *
	TMT[A]	46.2 (16.3)	31.1 (9.1)	*p* = 0.009 *
	TMT[B]	111.6 (32.5)	59.6 (14.7)	*p* < 0.001 *
	TMT[B-A]	65.4 (26.4)	28.5 (14.6)	*p* < 0.001 *
	TMT[B/A]	2.5 (0.7)	2 (0.6)	*p* > 0.05
	disease duration	10.5 (5.8)	5.2 (2.7)	*p* = 0.007 *
	UPDRS	17.3 (7.2)	14.0 (6.3)	*p* > 0.05

Sd values in brackets. *p*-values indicate differences of the parameters between the two clusters. Significant differences marked by *.

**Figure 5:**
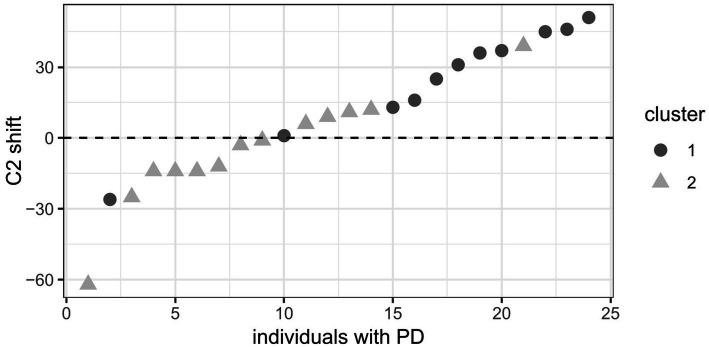
Clusters in the PD group ordered by C_2_ shift. Dark grey dots = cluster 1, light grey triangles = cluster 2. Positive C_2_ shift values (in milliseconds) indicate a rightward shift and negative values a leftward shift.

The two clusters within the PD group ordered by C_2_ shift behavior are visualized in Figure 5. It shows that speakers with PD in cluster 1 exhibit a more extreme rightward shift (dark grey dots) compared to those in cluster 2 (light grey triangles).

### 3.4 Correlation analysis

Correlations between shift patterns of C_2_ and the different TMT scores as well as disease duration and UPDRS were assessed for the PD group, excluding the outlier point, to further explain the mechanisms at play. The C_2_ shift correlates with all executive function scores (mental flexibility and set-shifting) involving TMT[B] (TMT[B], TMT difference [B-A], TMT ratio [B/A]). *p*-values and correlation coefficients for these correlations are presented in Table 6. Additionally, both C_2_ shift and all executive function scores (mental flexibility and set-shifting) involving TMT[B] correlate with disease duration (~ C_2_ shift: r_S_ = 0.654, *p* < 0.001 | ~ TMT[B]: r_S_ = 0.415, *p* = 0.044 | ~ TMT[B-A]: r_S_ = 0.558, *p* = 0.005 | ~ TMT[B/A]: r_S_ = 0.514, *p* = 0.010) while they do not correlate with UPDRS (*p* > 0.05 for all correlations). This indicates that more extreme rightward shifts of C_2_ as well as a decline in executive functioning are associated with longer disease duration but are independent of general motor impairment.

**Table 6:** *p-*values and correlation coefficients for the correlations of C_2_ shift and the TMT scores TMT[B], TMT[B-A], and TMT[B/A].

Condition	C2 shift ~ TMT[B]	C2 shift ~ TMT[B-A]	C2 shift ~ TMT[B/A]
med-ON	r_P_ = 0.423 *p* = 0.044	r_P_ = 0.540 *p* = 0.008	r_S_ = 0.526 *p* = 0.010

## 4 Discussion

This study looked at articulatory timing patterns of German syllable onsets that differ in complexity (CV vs. CCV) in HC and individuals with PD. For both groups, we found a leftward shift of C_1_ at the kinematic level, making room for C_2_ in C_1_C_2_V, as predicted by the C-center effect. Thus, the temporal interval between the C_1_ target position and the V target position is longer in clusters compared to singletons, indicating that the achievement of lower lip closure for /p/ occurs earlier relative to the vocalic target. For the rightmost C_2_, one would expect a shortening of the temporal interval between the C_2_ target position and the V target position, so that the target in CCV is achieved later than in CV syllables. However, we found no considerable rightward shift of C_2_ /l/, while, at the acoustic level, the segment is shortened in C_1_C_2_V. This indicates a non-symmetrical timing pattern as previously observed for the complex onset /pl/ in German (Brunner et al., 2014; Pouplier, 2012); C_1_ is moving to the left, while C_2_ remains at its position comparing CV and CCV syllable coordination. However, individuals with PD in med-OFF condition present with longer segment durations as well as longer latencies between consonants and vowels, while group differences are eliminated between HC and PD in med-ON. This finding lets us draw conclusions on the beneficial effect of levodopa on motor speech skills. Results of the executive function test show that the PD group presents with poorer performance scores when it comes to processing speed (TMT[A]) as well as cognitive flexibility (TMT[B]) and set-shifting skills (TMT[B-A]/ TMT[B/A]). For both groups, the cluster analysis revealed an age-dependent decline in executive functions which, in turn, correlated with a more extreme rightward shift of C_2_ for the PD group only. Additionally, for this group, the C_2_ shift and executive function scores both correlate with disease duration but not with UPDRS, hinting at a connection between ineffective articulatory timing and executive dysfunctions that is associated with longer disease duration.

Our results are in line with Brunner et al. (2014) and Pouplier (2012), as a non-symmetrical timing pattern for the complex onset coordination in /pl/ clusters is found for the neurotypical HC group. While the coarticulatory overlap between C_1_ /p/ and the vocalic target /i/ decreases as C_1_ shifts to the left (increase in temporal interval between articulatory targets), C_2_ /l/ does not shift toward the following vowel but slightly away from it from C_2_V to C_1_C_2_V. In line with Mücke et al. (2020) we confirm that the rightward shift of C_2_ is not required in /pl/ clusters in neurotypical German speakers. We can expect that the synergies between jaw, lips and tongue in the production of /pl/ lead to a biomechanical shortening effect of the tongue tip closure during the production of /l/, previously described by Mücke et al. (2020) for younger and older speakers. In other words: The tongue tip trajectory to the alveolar ridge during /l/ may be relatively short, potentially due to the already elevated jaw position during the preceding /p/. This coarticulatory effect is also revealed by shorter segmental durations of C_2_ on the acoustic surface as well lower peak velocities; a phenomenon that can be observed across all groups. The acoustic duration of /l/ in CV syllables is substantially longer in individuals with PD compared to HC, whereas this group difference is not present in the more complex CCV context. We assume that the mechanism of biomechanical shortening in CCV syllables accounts for the comparable /l/ durations across groups. This pattern likely results from changes in coarticulatory overlap, highlighting the influence of cognitive control on speech motor coordination. However, additional adjustments of intra-gestural timing patterns are also possible to preserve the C-center timing patterns in complex onsets.

This same non-symmetrical timing pattern is observed in the speech production of individuals with PD for complex syllable organization, even in a poor motor status, i.e., without medication. This means, in individuals with PD, C_1_ shifts to the left while C_2_ does not shift considerably to the right. As already mentioned, the C_2_ segment is also acoustically shortened in complex syllables for this group. The C_2_ shift patterns observed for both HC and individuals with PD in the present investigation all lie within the scope of what has been reported for neurotypical speakers. The across groups/conditions comparison reveals longer C_2_, V, and consecutively syllable durations for PD med-OFF compared to PD med-ON and HC. Accordingly, latencies from C_1_/C_2_ to V are longer in this condition as well. These results on inter-gestural timing patterns extend the findings of studies reporting stable and preserved timing patterns that are scaled proportionally in time in individuals with PD for vowel productions such as Yunusova et al. (2008). They also parallel those of Weiss et al. (1997) for limb movements in PD: Coordination of movements was found to be intact in terms of coordinative structures, but they take longer to be executed.

Looking at the differences within the PD group (med-OFF vs. med-ON), a beneficial effect of levodopa on speech planning abilities is found, which has already been detected before (e.g., Thies et al., 2021). Individuals with PD in med-OFF produce longer consonantal (C_2_ /l/) and vocalic (V /i/) movements. These durational changes at the intra-gestural level lead to longer latencies between Cs and *Vs* at the inter-gestural level in the med-OFF condition and are reduced under levodopa in the med-ON condition. Overall, a general trend of PD med-OFF > PD med-ON > HC emerges, where group differences between med-ON and HC are often eliminated.

Moving on to the neuropsychological test scores, processing speed (TMT[A]) and executive functions (mental flexibility – TMT[B], set-shifting – TMT[B-A] and [B/A]) seem to be impaired in PD even ON medication as patients presented with longer performance times compared to HC. However, we want to point toward a limitation of this study, as it might have in fact been beneficial to assess executive functions in the PD med-OFF condition despite the possible effects of motor deficits on task performance. The additional data points could have led to clearer group differences. This assumption is based on the results of studies such as Cools (2006) as well as Gul and Yousaf (2022) who found that levodopa intake improves performance on executive functioning/set-shifting tests in individuals with PD. We would therefore expect stronger group differences between HC and PD if cognitive abilities had been tested in med-OFF condition.

The results of the cluster analysis reveal an aging effect when it comes to the cognitive functions, as processing speed (TMT[A]) as well as executive functions (mental flexibility – TMT[B], set-shifting – TMT[B-A] and [B/A]) appear to decline with older age across groups. The articulatory shift patterns, however, are only affected in the PD group, where we find more extreme rightward shifts of C_2_ along with longer disease durations in addition to the aging effect. For the PD group, the C_2_ shift correlates with all executive functions scores as well as with disease duration, while it is independent of general motor severity as measured by the UPDRS. This relationship between timing patterns and executive functions, particularly mental flexibility and set-shifting, lets us assume that some individuals with PD change their articulatory timing patterns as C_2_ tends to shift more to the right when there is a decline in mental flexibility/set-shifting abilities, indicating the possibility of a less efficient/deviant timing. Due to the correlation between executive functions and disease duration, however, we cannot unequivocally eliminate the possibility of a parallel decline of articulatory coordination and executive functioning as the disease progresses. Future studies might want to disentangle this relationship further by looking at how much executive functions/disease duration independently relate to the C_2_ shift after controlling for the other. At the current state, the present investigation extends findings by Thies et al. (2020) who established a link between acoustic parameters of prominence marking and task-switching. It is yet to be determined, whether the observed interplay is due to executive functions declining more rapidly in PD and that the same pattern could consequently be observed in an older cohort of HC, or whether further non-motor deficits add on top of the impaired speech motor control in PD. Compensation strategies due to the underlying pathology in PD with the goal to maintain intelligible speech output might therefore differ from HC. Future research could test if HC speakers use similar compensation strategies that surface later, i.e., with older age. Within this context, it might also be relevant to consider that differences in compensation strategies between dysarthria in various pathologies (e.g., Amyotrophic lateral sclerosis) have been found (Weismer et al., 2003).

There are a couple of implications for speech therapy for individuals with PD, whether their symptoms are treated with levodopa or not, as speech therapy currently focuses mainly on the motor aspects of the disease (Smith and Caplan, 2018). The results underline that speech is inextricably linked to cognition and in the case of articulatory coordination even likely orchestrated by executive functions. The understanding that executive dysfunctions may be the cause of deviant coordination as a significant element of impaired articulation leads to the assumption that if cognitive executive training was included in speech therapy this would not only lead to improved executive skills but also alleviation of some of the speech symptoms in PD. A similar approach has been discussed for gait disorders in PD, as gait also depends on executive functions (Walton et al., 2018). Therapy outcomes would therefore be improved, majorly contributing to communication abilities which sit at the boundary between motor and non-motor impairment (Thies et al., 2020). The integrity of these abilities, in turn, has been shown to be a major factor in maintaining quality of life of the affected individuals (Miller et al., 2008). Taken together, the modified treatment options might alleviate some of the disease burden and improve quality of life.

A key limitation of the current study is the low number of repetitions per stimulus, with each participant producing each target utterance only twice. Particularly in kinematic studies examining speech in clinical populations using EMA, only a small number of repetitions (typically five) is commonly used (Kim et al., 2021; Hermes et al., 2019; Wong et al., 2011; Yunusova et al., 2010). The complexity of the experimental setup, participant fatigue, and overall recording burden due to other speech tasks constrained the number of feasible repetitions for our research. However, Parrell et al. (2017) report consistent coordination patterns across repetitions in individuals with motor speech disorders. Similarly, Sorensen and Gafos (2016) argue that even single productions can yield informative data. Based on this, we assume that speech coordination in our task is informative, particularly given the simplicity of the speech task we selected. We therefore consider our results to be meaningful for the research question under investigation. Additionally, the speech data analyzed here were recorded as part of a broader experimental protocol, which required distributing recording time across multiple speech tasks. It is also important to note that all recordings were conducted with individuals with PD in a state without any PD medication. Given the physical and cognitive challenges associated with this condition, it was essential to limit the duration of data collection to ensure participant comfort and compliance. This is also the reason why the TMT was only conducted in med-ON condition. Despite this, the study includes a relatively large sample size for EMA research, which supports the robustness and generalizability of group-level findings. Furthermore, coordination patterns across articulatory targets are generally stable and show minimal trial-to-trial variability within individuals. Nonetheless, the small number of repetitions may limit the detection of subtle intra-individual variability, and this should be taken into account when interpreting the results.

## Data Availability

Data files and R scripts are available from the corresponding author upon request.

## References

[ref1] Aarsland D. Batzu L. Halliday G. M. Geurtsen G. J. Ballard C. Chaudhuri K. R.. (2021). Parkinson disease-associated cognitive impairment. Nat. Rev. Dis. Primers 7:47. doi: 10.1038/s41572-021-00280-3, PMID: 34210995

[ref2] Bates D. Mächler M. Bolker B. Walker S. (2015). Fitting linear mixed-effects models using lme4. J. Stat. Softw. 67, 1–48. doi: 10.18637/jss.v067.i01

[ref3] Beck A. T. Steer R. A. Brown G. K. (1996). Manual for the Beck Depression Inventory-II. San Antonio, TX: Psychological Corporation.

[ref4] Bowie C. R. Harvey P. D. (2006). Administration and interpretation of the trail making test. Nat. Protoc. 1, 2277–2281. doi: 10.1038/nprot.2006.390, PMID: 17406468

[ref5] Browman C. P. Goldstein L. (2000). Competing constraints on inter-gestural coordination and self-organization of phonological structures. Bull. Commun. Parl. 5, 25–34.

[ref6] Brunner J. Geng C. Sotiropoulou S. Gafos A. (2014). Timing of German onset and word boundary clusters. J. Lab. Phonol. 5, 403–454. doi: 10.1515/lp-2014-0014

[ref7] Cools R. (2006). Dopaminergic modulation of cognitive function-implications for l-DOPA treatment in Parkinson's disease. Neurosci. Biobehav. Rev. 30, 1–23. doi: 10.1016/j.neubiorev.2005.03.024, PMID: 15935475

[ref8] Dajani D. R. Uddin L. Q. (2015). Demystifying cognitive flexibility: Implications for clinical and developmental neuroscience. Trends Neurosci. 38, 571–578. doi: 10.1016/j.tins.2015.07.003, PMID: 26343956 PMC5414037

[ref9] Domellöf M. E. Elgh E. Forsgren L. (2011). The relation between cognition and motor dysfunction in drug-naive newly diagnosed patients with Parkinson's disease. Mov. Disord. 26, 2183–2189. doi: 10.1002/mds.23814, PMID: 21661051

[ref10] Duffy J. R. (2019). “Motor speech disorders” in Substrates, Differential Diagnosis, and Management (4th ed.) (St. Louis, MO: Elsevier).

[ref11] Fahn S. Elton R. Committee, U. D (1987). “Unified parkinson’s disease rating scale” in Recent developments in Parkinson’s disease. eds. Fahn S. Marsden C. Calne D. Goldstein M., vol. 2 (Florham Park, NJ: Macmillan Healthcare Information), 153–163.

[ref12] Folstein M. F. Folstein S. E. McHugh P. R. (1975). “Mini-mental state”: A practical method for grading the cognitive state of patients for the clinician. J. Psychiatr. Res. 12, 189–198. doi: 10.1016/0022-3956(75)90026-6, PMID: 1202204

[ref13] Gul A. Yousaf J. (2022). Efficacy of l-dopa in treatment of aggression, frontal lobe cognitive functioning and task switching deficits in Parkinson’s disease patients. Pak. Armed Forces Med. J. 72, 132–135. doi: 10.51253/pafmj.v72iSUPPL-2.3354

[ref14] Hermes A. Mücke D. Auris B. (2017). The variability of syllable patterns in Tashlhiyt Berber and Polish. J. Phon. 64, 127–144. doi: 10.1016/j.wocn.2017.05.004

[ref15] Hermes A. Mücke D. Thies T. Barbe M. T. (2019). Coordination patterns in essential tremor patients with deep brain stimulation: syllables with low and high complexity. Lab. Phonol. 10:6. doi: 10.5334/labphon.141

[ref16] Iskarous K. Pouplier M. (2022). Advancements of phonetics in the 21st century: a critical appraisal of time and space in articulatory phonology. J. Phon. 95:101195. doi: 10.1016/j.wocn.2022.101195

[ref17] Jochim M. Winkelmann R. Jänsch K. Cassidy S. Harrington J. (2024). _emuR: Main Package of the EMU Speech Database Management System_. 2. Available online at:https://CRAN.R-project.org/package=emuR.

[ref18] Kalbe E. Calabrese P. Kohn N. Hilker R. Riedel O. Wittchen H.-U.. (2008). Screening for cognitive deficits in Parkinson's disease with the Parkinson neuropsychometric dementia assessment (PANDA) instrument. Parkinsonism Relat. Disord. 14, 93–101. doi: 10.1016/j.parkreldis.2007.06.008, PMID: 17707678

[ref19] Kalbe E. Rehberg S. P. Heber I. Kronenbuerger M. Schulz J. B. Storch A.. (2016). Subtypes of mild cognitive impairment in patients with Parkinson's disease: evidence from the LANDSCAPE study. J. Neurol. Neurosurg. Psychiatry 87, 1099–1105. doi: 10.1136/jnnp-2016-313838, PMID: 27401782

[ref20] Kassambara A. Mundt F. (2020). _factoextra: Extract and Visualize the Results of Multivariate Data Analyses_. R package version 1.0.7, Available online at:https://CRAN.R-project.org/package=factoextra>.

[ref21] Kim D. Kuruvilla-Dugdale M. de Riesthal M. Jones R. Bagnato F. Mefferd A. (2021). Articulatory correlates of stress pattern disturbances in talkers with dysarthria. J. Speech Lang. Hear. Res. 64, 2287–2300. doi: 10.1044/2021_JSLHR-20-00299, PMID: 33984259 PMC8740652

[ref22] Kühnert B. Hoole P. Mooshammer C. (2006) Gestural overlap and c-center in selected French consonant clusters. 7th international seminar on speech production (ISSP), 327–334

[ref23] Lenth R. (2024). _emmeans: Estimated Marginal Means, aka Least-Squares Means_. R package version 1.10.0. Available online at:https://CRAN.R-project.org/package=emmeans.

[ref24] McKinlay A. Grace R. C. Dalrymple-Alford J. C. Roger D. (2010). Characteristics of executive function impairment in Parkinson's disease patients without dementia. J. Int. Neuropsychol. Soc. 16, 268–277. doi: 10.1017/S1355617709991299, PMID: 20003582

[ref25] Miller N. Noble E. Jones D. Allcock L. Burn D. J. (2008). How do I sound to me? Perceived changes in communication in Parkinson's disease. Clin. Rehabil. 22, 14–22. doi: 10.1177/0269215507079096, PMID: 18089662

[ref26] Mücke D. (2018). “Dynamische Modellierung von Artikulation und prosodischer Struktur: Eine Einführung in die Artikulatorische Phonologie” in Studies in Laboratory Phonology4 (Berlin: Language Science Press).

[ref27] Mücke D. Hermes A. Tilsen S. (2020). Incongruencies between phonological theory and phonetic measurement. Phonology 37, 133–170. doi: 10.1017/S0952675720000068

[ref28] Muir R. T. Lam B. Honjo K. Harry R. D. McNeely A. A. Gao F. Q.. (2015). Trail making test elucidates neural substrates of specific poststroke executive dysfunctions. Stroke 46, 2755–2761. doi: 10.1161/STROKEAHA.115.009936, PMID: 26382176 PMC4589519

[ref29] Nip I. S. B. Burkem M. M. III Kim Y. (2023). The effects of deep brain stimulation on speech motor control in people with Parkinson’s disease. J. Speech Lang. Hear. Res. 66, 804–819. doi: 10.1044/2022_JSLHR-22-00443 36780302

[ref30] Parrell B. Agnew Z. Nagarajan S. Houde J. Ivry R. B. (2017). Impaired feedforward control and enhanced feedback control of speech in patients with cerebellar degeneration. J. Neurosci. 37, 9249–9258. doi: 10.1523/JNEUROSCI.3363-16.2017, PMID: 28842410 PMC5607467

[ref31] Pouplier M. (2012). “The gestural approach to syllable structure: universal, language- and cluster-specific aspects” in Speech planning and dynamics. eds. Fuchs S. Weirich M. Pape D. Perrier P. (Frankfurt am Main: Lang), 63–96.

[ref32] R Core Team (2024). _R: A Language and Environment for Statistical Computing_. Vienna, Austria: R Foundation for Statistical Computing.

[ref33] Reitan R. M. (1992). Trail making test: manual for administration and scoring. Tucson, AZ: Reitan Neuropsychology Laboratory.

[ref34] Rohl A. Gutierrez S. Johari K. Greenlee J. Tjaden K. Roberts A. (2022). Speech dysfunction, cognition, and Parkinson's disease. In Narayanan S. N. Albin R.L. (Eds.). Cognition in Parkinson's disease 269, 153–173. Amsterdam, Oxford, Cambridge: Elsevier 10.1016/bs.pbr.2022.01.017 PMC11321444 35248193

[ref35] Sánchez-Cubillo I. Periáñez J. A. Adrover-Roig D. Rodríguez-Sánchez J. M. Ríos-Lago M. Tirapu J.. (2009). Construct validity of the Trail Making Test: Role of task-switching, working memory, inhibition/interference control, and visuomotor abilities. J. Int. Neuropsychol. Soc. 15, 438–450. doi: 10.1017/S1355617709090626, PMID: 19402930

[ref36] Saranza G. Lang A. E. (2021). Levodopa challenge test: indications, protocol, and guide. J. Neurol. 268, 3135–3143. doi: 10.1007/s00415-020-09810-7, PMID: 32333167

[ref37] Smith K. M. Caplan D. N. (2018). Communication impairment in Parkinson’s disease: Impact of motor and cognitive symptoms on speech and language. Brain Lang. 185, 38–46. doi: 10.1016/j.bandl.2018.08.002, PMID: 30092448

[ref38] Sorensen T. Gafos A. (2016). The gesture as an autonomous nonlinear dynamical system. Ecol. Psychol. 28, 188–215. doi: 10.1080/10407413.2016.1230368

[ref39] Spencer K. A. Rogers M. A. (2005). Speech motor programming in hypokinetic and ataxic dysarthria. Brain Lang. 94, 347–366. doi: 10.1016/j.bandl.2005.01.008, PMID: 16098382

[ref40] Strauss E. Sherman E. M. S. Spreen O. (2006). A compendium of neuropsychological tests: administration, norms, and commentary. 3rd Edn. New York: Oxford University Press.

[ref41] Thies T. (2023). Tongue body kinematics in Parkinson’s disease: effects of Levodopa and deep brain stimulation. Berlin: Peter Lang Verlag.

[ref42] Thies T. Mücke D. Dano R. Barbe M. T. (2021). Levodopa-based changes on vocalic speech movements during prosodic prominence marking. Brain Sci. 11:594. doi: 10.3390/brainsci11050594, PMID: 34064356 PMC8147761

[ref43] Thies T. Mücke D. Lowit A. Kalbe E. Steffen J. Barbe M. T. (2020). Prominence marking in parkinsonian speech and its correlation with motor performance and cognitive abilities. Neuropsychologia 137:107306. doi: 10.1016/j.neuropsychologia.2019.107306, PMID: 31857118

[ref44] Walton C. C. Mowszowski L. Gilat M. Hall J. M. O'Callaghan C. Muller A. J.. (2018). Cognitive training for freezing of gait in Parkinson's disease: a randomized controlled trial. NPJ Parkinsons Dis. 4:15. doi: 10.1038/s41531-018-0052-6, PMID: 29796409 PMC5959878

[ref45] Weismer G. Yunusova Y. Westbury J. R. (2003). Interarticulator coordination in dysarthria: an X-ray microbeam study. J. Speech Lang. Hear. Res. 46, 1247–1261. doi: 10.1044/1092-4388(2003/097), PMID: 14575356

[ref46] Weiss P. Stelmach G. E. Hefter H. (1997). Programming of a movement sequence in Parkinson’s disease. Brain 120, 91–102. doi: 10.1093/brain/120.1.91 9055800

[ref47] Winkelmann R. Harrington J. Jänsch K. (2017). Emu-sdms: advanced speech database management and analysis in R. Comput. Speech Lang. 45, 392–410. doi: 10.1016/j.csl.2017.01.002

[ref48] Wong M. N. Murdoch B. E. Whelan B. M. (2011). Lingual kinematics in dysarthric and nondysarthric speakers with Parkinson's disease. Parkinsons Disease 2011:352838, 1–8. doi: 10.4061/2011/352838, PMID: 22007341 PMC3191771

[ref49] Yunusova Y. Green J. R. Lindstrom M. J. Ball L. J. Pattee G. L. Zinman L. (2010). Kinematics of disease progression in bulbar ALS. J. Commun. Disord. 43, 6–20. doi: 10.1016/j.jcomdis.2009.07.003, PMID: 19683250 PMC2813314

[ref50] Yunusova Y. Weismer G. Westbury J. R. Lindstrom M. J. (2008). Articulatory movements during vowels in speakers with dysarthria and healthy controls. J. Speech Lang. Hear. Res. 51, 596–611. doi: 10.1044/1092-4388(2008/043), PMID: 18506038

[ref51] Ziegler W. Vogel M. (2010). Dysarthrie verstehen-untersuchen-behandeln. Stuttgart: Georg Thieme Verlag.

