# Supplementary material for: Executive dysfunctions impair and levodopa improves articulatory timing in Parkinson‘s disease
Source: Front Hum Neurosci. 2025 Jul 2;19:1580376. doi: 10.3389/fnhum.2025.1580376 (PMC12263582; doi:10.3389/fnhum.2025.1580376)
Supplement: Supplementary file 1 [file Table_1.pdf]

Overview of demographic and clinical data of individuals with PD and HC.

PD:

ID	sex	age (years)	UPDRS (OFF)	UPDRS (ON)	disease duration (years)	TMT[A]	TMT[B]	TMT[B-A]	TMT[B/A]	BDI	PANDA	MMST
PD1	m	69	48	17	13	77.54	147.76	70.22	1.91	0	23	24
PD2	w	51	23	12	6	28.20	52.20	24.00	1.85	6	30	30
PD3	m	61	54	16	19	60.23	113.97	53.74	1.89	9	25	29
PD4	m	63	15	12	10	38.02	131.86	93.84	3.47	11	23	29
PD5	m	54	22	6	5	33.29	59.16	25.87	1.78	6	22	29
PD6	m	63	48	28	6	54.82	179.60	124.78	3.28	1	25	29
PD7	w	56	45	25	6	53.27	63.72	10.45	1.20	0	27	30
PD8	m	68	39	6	12	34.81	87.81	53.00	2.52	3	24	29
PD9	m	56	16	12	5	32.99	61.08	28.09	1.85	0	14	24
PD10	w	70	22	7	20	28.10	102.00	73.90	3.63	9	14	28
PD11	m	62	46	24	8	40.12	88.22	48.10	2.20	9	28	28
PD12	m	58	20	11	2	42.50	65.00	22.50	1.53	7	26	30
PD13	m	53	29	14	4	27.80	43.40	15.60	1.56	5	27	29
PD14	w	69	27	14	13	27.03	57.45	30.42	2.13	10	29	29
PD15	m	79	31	26	2	54.02	103.71	49.69	1.92	17	24	29
PD16	m	56	30	15	1	21.24	32.20	10.96	1.52	3	27	29
PD17	m	65	36	20	10	32.33	108.00	75.67	3.34	23*	26	30
PD18	m	65	20	20	3	61.00	107.00	46.00	1.75	5	27	29
PD19	m	60	48	17	8	23.21	49.20	25.99	2.12	10	25	29
PD20	m	42	34	19	7	28.27	88.44	60.17	3.13	3	24	30
PD21	m	59	23	7	4	27.79	50.04	22.25	1.80	4	20	30
PD22	m	56	14	9	7	31.53	79.20	47.67	2.51	12	14	27
PD23	w	56	9	9	2	19.09	64.10	45.01	3.36	8	26	29
PD24	m	66	45	26	11	35.46	67.06	31.60	1.89	1	19	28
PD25	m	54	44	27	6	56.41	344.00	287.18	6.10	15	18	30
**mean (sd)**	5 f 20 m	60 (7)	31 (12)	16 (7)	8 (5)	38.8 (14.9)	94.1 (61.6)	55.3 (54.4)	2.42 (1.03)	7 (6)	24 (5)	29 (2)

* Study participation of this patient was approved after a check-in with a psychiatrist.

HC:

ID	sex	age (years)	UPDRS	TMT[A]	TMT[B]	TMT[B-A]	TMT[B/A]	BDI	PANDA	MMST
HC1	m	72	6	35.01	68.62	33.61	1.96	4	25	28
HC2	m	66	5	37.13	73.23	36.10	1.97	4	15	29
HC3	m	52	NA	24.26	58.41	34.15	2.41	2	25	28
HC4	w	67	5	31.24	91.02	59.78	2.91	2	27	28
HC5	m	58	1	24.82	69.45	44.63	2.80	0	26	30
HC6	w	48	3	34.31	68.67	34.36	2.00	0	29	29
HC7	m	64	12	19.60	62.63	43.03	3.20	2	29	29
HC8	m	45	0	13.56	38.04	24.48	2.81	7	27	30
HC9	m	55	1	22.28	55.97	33.69	2.51	0	30	29
HC10	m	53	2	24.78	59.64	34.86	2.41	0	19	29
HC11	w	57	4	24.25	40.05	15.80	1.65	1	30	30
HC12	m	67	6	27.15	114.75	87.60	4.23	0	18	28
HC13	m	57	6	23.20	91.52	68.32	3.94	0	22	29
HC14	m	50	2	22.18	50.13	27.95	2.26	3	24	30
HC15	m	79	NA	NA	NA	NA	NA	3	24	30
HC16	m	61	16	55.73	118.45	62.72	2.13	3	26	29
HC17	m	62	5	43.34	84.77	41.43	1.96	1	20	29
HC18	w	71	7	37.00	90.81	53.81	2.45	0	26	28
HC19	m	57	5	50.05	62.24	12.19	1.24	0	30	28
HC20	m	72	1	34.89	173.24	138.35	4.97	0	28	29
HC21	w	59	NA	24.96	76.50	51.54	3.06	5	24	30
	5 f 16 m	61 (9)	5 (4)	30.3 (10.2)	78.1 (31.3)	47.8 (28.4)	2.67 (0.9)	2 (2)	25 (4)	29 (1)
